# Dr. Newsholme, Medical Officer of the Local Government Board

**Published:** 1908-01-25

**Authors:** 


### THE MEDICAL OFFICER OF THE LOCAL GOVERNMENT BOARD.

Another medical appointment to a principal Government office, which reflects the greatest credit upon the Minister responsible for the selection, has to be recorded. Dr. Newsholme's appointment as Chief Medical Officer to the Local Government Board is a fitting complement to that of Dr. Newman as Medical Adviser to the Board of Education. In both instances administrative officers of proved ability, and a reputation based upon the acknowledged quality of their work, have been appointed to those central Government offices to which their experience in civic medicine unmistakably has pointed. Dr. Newsholme will take with him to the Local Government Board a reputation which has been established as much by his unceasing industry and indefatigable energy as by the ability which he has brought to bear on the many problems of health to which he has addressed himself. Primarily a man of medicine, he is an eminent statistician, an epidemiologist of repute, a social reformer scientifically informed from his approach to sociology through the gateway of State medicine, and a voluminous contributor on innumerable topics relating to public health. Among medical officers of health he is of the school which has kept alive to the broad and important aims which have ever transcended the immediate routine of the municipal official. The problems of health have grown apace, and far-reaching and momentous changes in medical organisation are pending the new developments in public health administration. The nation is awakening to the importance of health; it is learning how seriously it is handicapped by the menacing encroachments made upon this primal national need, and the time of action has arrived when fresh dispositions are being made to check and reduce the vast amount of preventable disease which it is now established exists. At such a time there is need that the chief medical advisers to the departments of State principally concerned should be men of ability, profoundly in sympathy with the great movements which in large measure they will guide. We think that the public and the medical profession are to be congratulated in having two such men as Dr. Newsholme and Dr. Newman to advise the Local Government Board and the Board of Education when the important changes necessitated by existing and prospective public health legislation are in process. We are mindful that the report of the Royal Commission on the Poor-laws, when it is issued, is likely to produce as great legislative changes as those which followed the Inter-Departmental Report on Physical Deterioration, and if we are right in this anticipation it will be apparent how important it is that the office to which Dr. Newsholme is appointed should be filled by a capable administrator fully in sympathy with the prospective changes.

Dr. Newsholme is Medical Officer of Health of Brighton, President of the Epidemiological Section of the Royal Society of Medicine, and Past-President of the Incorporated Society of Medical Officers of Health, Editor of *Public Health*, and Joint-Editor of *Hygiene*. His work on Vital Statistics is probably the best-known book on this important branch of scientific medicine, while his contributions to the epidemiology of diphtheria, rheumatic fever, and consumption have been suggestive and valuable.

While we have felt constrained to differ from many of the views with which he is identified, we are pleased to acknowledge that his attainments and his medical and administrative experience distinguish him as eminently suited to the office to which he has been called.

We do not doubt that in this wider sphere of work he will maintain the reputation he has established. The fact that he has been a medical officer of health and has had direct personal experience of the difficulties which beset that office, and of the many irritating and disabling circumstances which curtail its usefulness, will not be the least of the valuable experiences he will bring to his new duties.

